# Advancing Semiochemical Tools for Mountain Pine Beetle Management: *Dendroctonus ponderosae* Responses to Saprophytic Fungal Volatiles

**DOI:** 10.3390/metabo15070488

**Published:** 2025-07-20

**Authors:** Leah Crandall, Rashaduz Zaman, Guncha Ishangulyyeva, Nadir Erbilgin

**Affiliations:** Department of Renewable Resources, University of Alberta, Edmonton, AB T6G 2H1, Canada; lccranda@ualberta.ca (L.C.); rashaduz@ualberta.ca (R.Z.); ishangul@ualberta.ca (G.I.)

**Keywords:** bark beetles, forest health, insect behaviour modulation, microbial emissions, population monitoring, volatile-mediated interaction

## Abstract

Background/Objectives: Within their host trees, mountain pine beetles (MPBs, *Dendroctonus ponderosae*) interact with many fungal species, each releasing a unique profile of volatile organic compounds (VOCs). The FVOCs released by the two primary symbionts of MPBs, *Grosmannia clavigera* and *Ophiostoma montium*, have been found to enhance MPB attraction in the field and laboratory studies. Opportunistic, saprophytic fungal species, such as *Aspergillus* sp. and *Trichoderma atroviride*, are also common in MPB galleries and can negatively impact MPB fitness. However, little is known about the FVOCs produced by these fungal species and how they may impact MPB feeding and attraction. Methods: To address this knowledge gap, we characterized the FVOC profile of *T. atroviride*, and performed bioassays to test the effects of its FVOCs on MPB attraction and feeding activity. Results: Our chemical analysis revealed several FVOCs from *T. atroviride* known to inhibit the growth of competing fungal species and impact subcortical-beetle attraction. Conclusions: From those FVOCs, we recommended four compounds—2-pentanone, 2-heptanone, 2-pentanol, and phenylethyl alcohol—for use in future field tests as anti-attraction lures for MPBs. In bioassays, we also observed strong MPB repellency from FVOCs released by *T. atroviride*, as well as the mild effects of FVOCs on MPB feeding activity. Our findings highlight the potential for these FVOCs to be utilized in the development of more effective MPB anti-attractant lures, which are crucial for the monitoring and management of low-density MPB populations.

## 1. Introduction

To successfully survive and reproduce within their selected host trees, mountain pine beetles [MPBs, *Dendroctonus ponderosae* (Hopkins), Coleoptera: Curculionidae, Scolytinae] depend on the presence of their symbiotic, ophiostomatoid fungi, primarily *Grosmannia clavigera, Ophiostoma montium*, and *Leptographium longiclavatum*. After entering their host tree, MPBs begin to feed on the phloem tissues and excavate egg galleries. During this process, beetles also inoculate trees with their symbiotic fungi, which are transported in beetle’s mycangia. Once entrance wounds are made in the tree by adult MPBs, opportunistic, saprophytic fungal species, such as *Aspergillus* sp. and *Trichoderma atroviride*, are also able to enter the beetle galleries and colonize the host tissues. Beetle interactions with both symbiotic and saprophytic fungal species are known to significantly impact beetle fitness and survival in their host [1,2,3,4]. Currently, we have limited understanding of the mechanisms underlying the complex interactions among the saprophytic fungi, the symbiotic fungi, and MPBs.

These ophiostomatoid and saprophytic fungi found in host trees release different concentrations and compositions of fungal volatile organic compounds (FVOCs), which are known to influence interactions between MPB, fungi, and other organisms [5,6,7]. Previous work from Zaman et al. (2023) [6] found a strong MPB attraction to volatiles released by *G. clavigera* and *O. montium* in olfactometer bioassays. In these bioassays, 80% of beetles chose these fungal treatments over the control (fungus-free) treatment. Further field experiments tested the MPB attraction to the most abundant FVOCs associated with its symbiotic fungi, with and without the MPB pheromone lures, and found two possible synergistic FVOCs, 2-methyl-1-butanol and 2-methyl-2-butanol [7].

Overall, the presence of symbiotic fungi in host tissues is known to increase beetle fitness through several mechanisms, including metabolizing host defence compounds, concentrating host tree nutrients during larval feeding, and acting as an additional food source for beetles [2,3,4,8]. Briefly, larval feeding preference for phloem colonized by *O. montium* and *G. clavigera* over un-colonized phloem has been attributed to the fungi acquiring and concentrating nitrogen from sapwood tissues and making more dietary nitrogen available to larvae [2,8]. The presence of *G. clavigera* has also been found to increase beetle gallery lengths and weight in bioassays using media amended with host monoterpenes [9]. This increase in MPB fitness is likely attributed to MPB fungal symbionts converting chemicals harmful to beetles to less toxic oxygenated monoterpenes [6,9]. Beetles emerging from trees infected with their symbionts, *G. clavigera* and *O. montium*, have also been found to be larger, with shorter development times and higher brood production [2]. Finally, adult MPBs which did not feed directly on conidia of symbionts were also found to be less likely to produce egg galleries and lay eggs [10].

Not all fungi found in bark beetle larval galleries are beneficial to adult bark beetles or their larvae; for instance, saprophytic fungi can reduce the length of ovipositional galleries, the length of larval galleries, and the number of beetle larvae by competing with MPBs and their symbiotic fungi [1,4]. In particular, *Aspergillus* sp. and *Trichoderma* sp. are common saprophytic fungi found in bark beetle galleries. They have been widely studied as biocontrol agents, and their FVOCs are known to suppress the mycelial growth of other fungal species [11,12]. The FVOCs emitted by *Trichoderma* sp. have also been found to mediate plant growth and induce plant resistance to pathogens [13]. Currently, it is unknown whether the FVOCs of antagonistic, saprophytic species may also act as semiochemical cues for MPBs, impacting beetle attraction to their aggregation pheromones. Additionally, to our knowledge, no previous studies have investigated the direct effects of FVOCs emitted from symbiotic and saprophytic fungi on MPB feeding behaviour. Understanding how FVOCs released by fungi present in MPB galleries influence both MPB attraction and feeding behaviour is essential for elucidating MPB–fungal interactions and could help clarify the mechanisms underlying the reduction in bark beetle fitness observed in the presence of saprophytic fungi in galleries.

Semiochemicals are highly effective and environmentally friendly tools for managing bark beetles, demonstrating successful population reduction across various species [14]. The intricate dynamics of bark beetle host location and colonization hinge on a delicate balance between attractant and anti-attractant compounds [15]. Notably, among anti-attractant compounds identified for MPBs, verbenone emerges as a single compound that has been found to effectively disrupt MPB attraction to their pheromones (Table 1). However, verbenone has also been found to be ineffective in some applications, depending on beetle population densities, the duration of verbenone use in a forest area, and diameters of available host trees in managed stands [16]. Given these limitations, FVOCs offer promising potential to complement verbenone by introducing additional, ecologically relevant cues that may enhance repellency or disrupt host location behaviours.

Conventional methods, particularly pull systems, primarily rely on attractants and have been extensively employed for MPB management. However, the emergence of push–pull systems presents a promising avenue for future MPB management strategies, particularly for beetles at low densities [14]. In contrast to pull systems operating alone, push–pull systems involve a coordinated interplay of attractants and anti-attractants, showcasing a remarkable efficacy in reducing MPB populations, with reported reductions as high as 69% [14]. The most potent push–pull systems strategically incorporate a combination of non-host volatiles (such as FVOCs) and species-specific and non-species-specific semiochemicals as anti-attractants. This synergistic approach has proven exceptionally effective, resulting in a staggering 92% reduction in bark beetle populations, including MPBs [14]. In comparison, reliance solely on species-specific semiochemicals, such as verbenone, has demonstrated lesser efficacy, with population reductions capped at 52% [14]. To advance the development of a robust push–pull management strategy for MPBs, it is imperative to dedicate further efforts to the identification and testing of potent anti-attractant chemicals beyond verbenone that may work alone or synergistically with verbenone to repel MPB.

Given known MPB attraction to FVOCs released from symbiotic fungi, the objective of our experiments was to first characterize the volatiles released by *Trichoderma atroviride*, a saprophytic species commonly found in MPB galleries, and then to determine whether the FVOCs released by this fungus could act as a repellent in 2-choice olfactometer assays. In application, the goal of this work is to identify anti-attractant compounds for MPBs that could be components of a more complex anti-attraction blend, in addition to verbenone. This would contribute to developing a more effective pull-push monitoring system for low-density MPB populations, which is crucial for monitoring and managing MPB populations on the frontier of range expansions.

In addition to the effects of FVOCs emitted by saprophytic fungi on MPBs during primary host selection, FVOCs may also influence MPB behaviour once they have entered their host. Despite no previous experiments testing the effects of FVOCs on MPB feeding behaviour, several studies have investigated the effects of fungal presence on MPB feeding. In bioassays examining the differences in tunnelling behaviours of *Ips typographus*, Kandasamy et al. (2023) [27] found that media colonized by symbiotic fungal species increased the tunnelling length and tunnelling probability of beetles after 48 h compared to media colonized by saprophytic fungus, *Trichoderma* sp. They suggest that the blends of oxygenated monoterpenes produced by symbionts from host tree monoterpenes likely direct beetles toward breeding or feeding sites. Due to fungi metabolizing and reducing the toxicity of host defence compounds for beetles, it is possible that synchronizing beetle feeding sites with areas of symbiotic fungal growth may increase beetle fitness [28]. Cardoza et al. (2006) [1] observed that adult *Dendroctonus rufipennis* behaviour changed in response to the presence of saprophytic fungi, including *Trichoderma* sp. Beetles either abandoned infested galleries or used fecal pellets to create physical barriers between them and the saprophytic fungus [1]. Another feeding bioassay, which measured tunnelling lengths of *Dendroctonus frontalis* larvae in media colonized by antagonistic fungal species found that larvae avoided the fungus by creating long tunnels [29]. This effect was attributed to the indirect effect of antagonistic fungal competition with symbiotic fungi. However, since this experiment focused on larval response to fungi, it may not represent adult bark beetle feeding behaviour [28].

Overall, the effects of FVOCs on MPB feeding and feeding site selection remainunclear: it is unknown whether the FVOCs released by saprophytic species may harm beetle fitness directly or indirectly by reducing the growth of symbiotic fungi. If saprophytic FVOCs directly impact beetle fitness, we would expect a reduction in overall beetle tunnelling when exposed to those volatiles compared to FVOCs from symbiotic species. While some studies have tested the interactions between bark beetle feeding on media colonized by different fungal species, to our knowledge, no studies have tested the effects of FVOCs alone on the feeding activities of MPBs. Therefore, the effects of fungi and their FVOCs on MPB feeding are still largely unknown. To test the effect of fungal volatiles emitted by a saprophytic fungus, *T. atroviride*, on the feeding behaviour of MPBs, we developed a novel feeding bioassay setup. We tested the effects of volatiles emitted from two isolates of *T. atroviride* compared to *G. clavigera* and a fungus-free control on the feeding activity of MPBs.

## 2. Materials and Methods

### 2.1. Insect Rearing

Hundreds of live adult MPBs were collected from naturally infested lodgepole pine (*Pinus contorta*) trees near Cranbrook, British Columbia (49°21′10″ N, 115°32′12″ W), in September 2024. Populations of MPB in the Cranbrook area were at low ensity at the time trees were harvested. Trees were felled and cut into 30 cm long bolts, which were then waxed on the cut ends to prevent desiccation. Beetles from the collected bolts were reared in plastic containers at room temperature near a bright light. Glass jars were placed at the base of the containers to collect emerging beetles. Adult beetles began to emerge after approximately one month and were kept in falcon tubes with a damp paper towel at 4 °C. Beetles were stored for a maximum of 3 days between emergence and use in bioassays. Active beetles were randomly selected for bioassays.

### 2.2. Fungal Isolates and Media Preparation

Master cultures of all fungal isolates used in our experiments were grown on potato–dextrose agar (PDA) media. After seven days of growth, in darkness and at room temperature, plugs from the actively growing margin of the plates were used for subculturing. For bioassays and volatile collections, we used a plant-based media modified from Wallin and Raffa (2000) [30] that consisted of PDA mixed with phloem and sapwood powder to grow the fungal isolates [9,31]. The PDA media was prepared according to manufacturer directions: 24 g PDA powder mixed in 600 mL of distilled water (Becton, Dickinson and Company, Mississauga, ON, Canada). Sections of phloem and sapwood were collected from healthy lodgepole pine bolts and dried in the oven at 50 °C for 72 h. Dried tissues were then ground using TissueLyser II (Qiagen, Montreal, QC, Canada) and autoclaved at 121 °C for 20 min to remove any remaining volatile compounds such as monoterpenes and sesquiterpenes and to sterilize the powder. Subsequently, 40 g of phloem and sapwood powder at a 9:1 ratio was added to 600 mL of liquid media (PDA and distilled water). The PDA media was then autoclaved at 121 °C for 20 min, and 7.5 mL was poured into small petri plates (60 mm diam. × 5 mm height) (Fisher Scientific, Toronto, ON, Canada). Media used to grow the fungal isolates can affect both the volatiles produced (composition and concentration) and the growth rate of fungi [32,33]. Adding phloem and sapwood powder to the media mimics the natural growth conditions of the fungi in host trees, where fungi grow less quickly but accumulate more biomass than PDA alone. Using the same MPB diet media in all our experiments also allowed us to identify the volatiles MPBs were interacting with in bioassays through our volatile extraction experiment.

We used three different fungal isolates throughout all our experiments: (1) *T. atroviride isolate* YL145, (2) *T. atroviride* isolate 1416, (3) *G. clavigera* isolate EL035. DNA was extracted and amplified using ITS1F (Forward Primer Sequence: CTTGGTCATTTAGAGGAAGTAA) [34,35] and ITS4 (Reverse Primer Sequene: TCCTCCGCTTATTGATATGC) [35,36] primers to confirm the species of fungal isolates. Sanger sequencing was performed by the Molecular Biology Facility (MBSU) at the University of Alberta, and sequence data were analyzed using Geneious Prime Version 2024.0.4 (GraphPad Software LLC d.b.a. Geneious (Boston, MA, USA)). The DNA of the *G. clavigera* isolate was not extracted, however, this specific isolate has been used in previous laboratory experiments and was originally collected from naturally MPB-infected galleries in Alberta [9,37]. The *T. atroviride* isolate YL145 was isolated from the phloem of lodgepole pine collected in the Edson Forest Area, Alberta, Canada (53.378° N, 115.4861° W). Due to differences in growth rates of fungal isolates, fungi were grown on plates until their mycelia occupied approximately 70% of the Petri plates; see Table 2 for the number of days each fungus was grown on each solid media type before being used in experiments. All fungi were grown in the darkness and at room temperature. *Grosmannia clavigera* was grown solely on PDA supplemented with phloem and sapwood.

### 2.3. Volatile Collection and Extraction

To characterize the volatile profile released by *T. atroviride*, we collected the volatiles from one isolate of the fungus grown on: (1) MPB diet media (phloem & sapwood mixed with PDA), and (2) PDA media without any amendments. Our experiments also included fungus-free control treatments for each media type. Headspace volatile collection and extraction methods were modified from methods described earlier [5,6,38]. Petri dishes containing media and fungal treatments were placed in 473 mL glass volatile collection chambers with Teflon tape on the threading and sealed with metal lids. A vacuum/pressure pump (Cole-Parmer Canada Inc., Montreal, QC, Canada) was attached to the lids of the jars. Fungal treatments were placed in the jars for 24 h to allow volatiles to accumulate, then, a constant airflow of 450 mL min^−1^ was set through the chamber lines for 24 h using a flowmeter. Teflon tubes filled with activated carbon (450 mg; 6–14 mesh Fisher Sci., Hampton, NH, USA) and secured with glass wool on either end were used to collect headspace volatiles. At the end of the 24 h collection period, collection tubes were removed from the setup, sealed with parafilm, wrapped in aluminum foil, and stored at −40 °C for no more than two days prior to chemical extraction, following our earlier work [6,37].

For the volatile extraction, an activated carbon sample was added to a microtube containing 1 mL of dichloromethane with tridecane as the internal standard (0.001%). The solution was vortexed for 30 s, sonicated for 10 min, and centrifuged (at 12,700 rpm) for 30 min before 1 mL of the separated organic phase was transferred to a 2 mL glass gas chromatography (GC) vial. This procedure was repeated twic for each sample [5]. Ten replicates were collected for each unique combination of fungal isolate and solid media, for a total of 48 samples (1 isolate and 1 control × 2 solid media types × 12 replicates).

### 2.4. Two-Choice Olfactometer Bioassays

To test the effect of VOCs emitted by saprophytic fungi on the attraction of MPBs, we conducted a bioassay using a two-choice olfactometer design previously described by Zaman et al. (2023) [6]. In olfactometer assays, we tested combinations of four treatments: (1) *T. atroviride* isolate YL145, (2) *T. atroviride* isolate 1416, (3) *G. clavigera* isolate EL035, and (4) a fungus-free control. These treatments were used to (1) determine whether VOCs emitted by *T. atroviride* act as repellents for MPB and (2) compare MPB attraction to VOCs released from antagonistic vs. symbiotic (*G. clavigera*) fungi. We first tested one isolate of *G. clavigera* against the control treatment (n = 20) to determine if the olfactometer setup produced results similar to those of Zaman et al. (2023) [6]. Our results were consistent with these findings (85% at *p* < 0.01). We then tested each isolate of *T. atroviride* against the fungus-free control to determine MPB repellency to saprophytic fungal volatiles (n = 20). We also tested each isolate of *T. atroviride* against the *G. clavigera* isolate (n = 20) to understand how beetles would interact with fungal volatiles of saprophytes and ophiostomatoid fungi when tested simultaneously.

**Figure 1:**
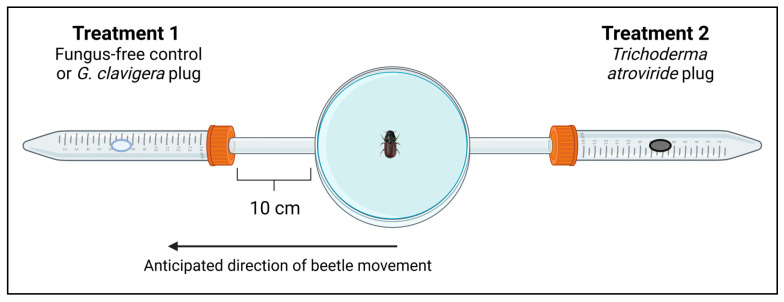
Two-choice olfactometer setup used in volatile choice experiment, designed by Zaman et al. (2023) [6]. Treatments included (1) *Trichoderma atroviride* isolate YL145, (2) *T. atroviride* isolate 1416, (3) *Grosmannia clavigera*, and (4) a fungus-free control. All treatments were grown on mountain pine beetle diet media. One 6 mm treatment plug was placed in either falcon tube and beetle choice was recorded after 20 min. Figure created in https://BioRender.com (accessed on 30 May 2025).

The olfactometer setup was identical to the one used by Zaman et al. (2023) [6] and consisted of a small petri dish (60 mm diam. × 15 mm height) (Fisher Scientific) with two polyvinyl chloride tubes (10 cm long) attached at opposite sides (Figure 1). The other end of each tube was attached to a 15 mL Falcon tube through a hole created in the lid. The tubes provided a pathway for beetles to move from the petri dish to the Falcon tubes, which contained fungal treatments. A circular piece of filter paper was placed in the petri dish to provide traction for beetle movement. Since MPBs are positively phototactic insects, the petri dish was also covered in black electrical tape to encourage movement towards one of the falcon tubes. A 6 mm plug of one of our treatments was placed in each falcon tube. The plugs were placed in the olfactometer setup one hour prior to the experiment to allow volatiles to accumulate. One female MPB was placed in the center of the petri dish. The beetle choice was determined by which tube the beetle was located in after 20 min had elapsed. Beetles that did not make a choice after 20 min were recorded and replaced with a different beetle.

### 2.5. Feeding Bioassays

**Figure 2:**
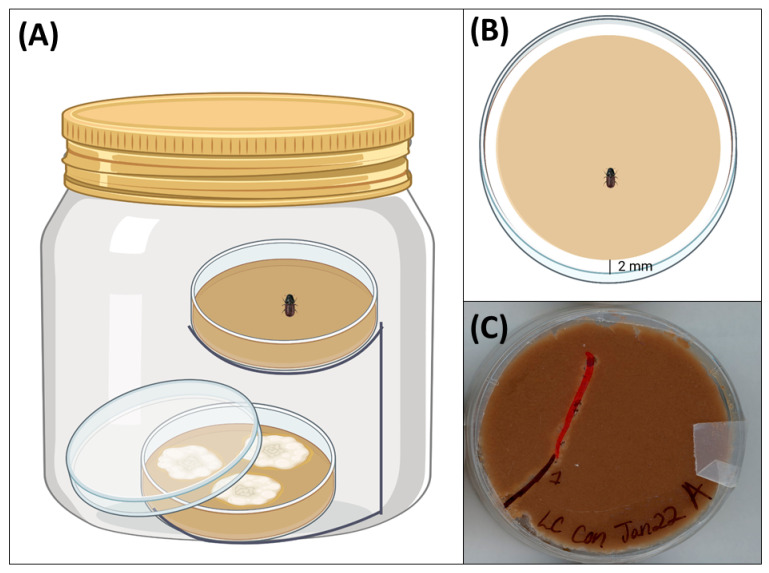
Feeding bioassay setup: (**A**) A glass bioassay chamber containing layered Petri dishes with fungal treatment (bottom) and fungus-free mountain pine beetle (MPB) diet media with a female MPB (top). (**B**) The top view of the upper Petri dish contains MPB diet media with a 2 mm gap between the media and the edge of the dish. (**C**) The underside view of MPB tunnelling in a petri dish after 48 h. Diagrams created in https://BioRender.com(accessed on 30 May 2025). Note: diagrams are not to scale.

The feeding bioassay consisted of two stacked Petri dishes (one at the bottom and one on top) placed together in a glass bioassay chamber (Figure 2). The bottom Petri dish contained a fungal treatment grown on MPB diet media, and the top Petri dish contained fungus-free MPB diet media. We wanted to observe whether the volatiles released from the fungal treatments in the bottom dish would impact MPB feeding in the top dish. We used wire stands to layer the petri dishes in the bioassay jars. The lid of the bottom dish was placed at an angle to allow fungal volatiles to escape, and the bottom dish was placed approximately 6 cm from the top petri dish. To avoid bacterial contamination due to excess moisture in the top dish, we allowed the media to dry under a fume hood for 3 h at room temperature. After drying, the media was inverted onto the lid to create a 2 mm space between the media and the edge of the dish. encouraging beetle tunnelling. Nine holes (1 mm dia) were made in another Ptri dish lid. A single female MPB was placed in the center of the media, and the lid with holes was secured to the lid containing media using parafilm.

Fungal treatments were placed at the bottom of the bioassay chambers for 4 h before adding the Petri dishes containing MPBs to allow volatiles to accumulate in the jars. Once the petri dish containing MPBs was placed in the bioassay chambers, the lids were secured with parafilm. Chambers were kept at room temperature for a total of 48 h. Beetle tunnelling activity in the media was measured at 24 and 48 h. ImageJ software (v1.54g, National Institutes of Health, Bethesda, MD, USA) was used to quantify the total tunnelling length, length of the longest continuous tunnel segment and the number of branches coming off of the longest tunnel segment at 24 and 48 h.

### 2.6. Statistical Analyses

The metabolomics data analysis platform Metaboanalyst 6.0 was used for visualization and multivariate statistical analysis of the final chemical concentration data from our FVOC collection experiment (http://www.metaboanalyst.ca, accessed on 1 April 2025). The data were autoscaled and log-transformed to obtain a normal distribution. The Euclidean distance measure and Ward clustering method were used in the analysis. Only volatile chemicals previously identified as fungal volatiles were included in the analysis.

R-studio (version 2022 4.2.1) was used to perform the remainder of the statistical analyses. To determine beetle attraction or repulsion to treatments tested in two-choice olfactometer bioassays, we performed a Chi-Square Goodness-of-Fit Test for each treatment pair tested. The null hypothesis assumed that beetles exhibit no preference, selecting either side of the bioassay chamber with equal probability (50:50 distribution). Expected frequencies were set accordingly, and the significance threshold was set at α = 0.01 in statistical tests.

R-studio (version 2022 4.2.1) was used to perform all statistical analyses for feeding bioassay data. To identify significant differences in MPB feeding behaviour across treatments, we performed Kruskal–Wallis rank-based tests, a non-parametric alternative to ANOVA, which is suitable for non-normally distributed data. We included multiple variables reflecting the beetle feeding behaviour: the total tunnelling length, the length of the longest continuous tunnel segment, and the number of branches coming off the longest continuous tunnel segment. Tunnelling was measured at 24 and 48 h for each replicate. Beetles that did not tunnel were excluded from all analyses except for the total tunnelling length analysis, where their tunnelling length was recorded as zero. For variables that differed significantly among treatments (*p* < 0.05), we performed pairwise comparisons using Dunn’s post hoc test with Bonferroni’s *p*-value adjustment to control for multiple comparisons. To compare the number of beetles that did not tunnel in each treatment, we constructed a 2 × 3 contingency table. Due to small sample sizes and the presence of low expected frequencies (<5) in more than 20% of cells, we used Fisher’s Exact Test with Monte Carlo approximation (10,000 simulations) to determine whether the distribution of non-tunnelling beetles differed significantly among treatments. Tests were two-tailed, and significance was set at α = 0.05.

## 3. Results

### 3.1. Volatiles Identified from Trichoderma Atroviride

**Figure 3:**
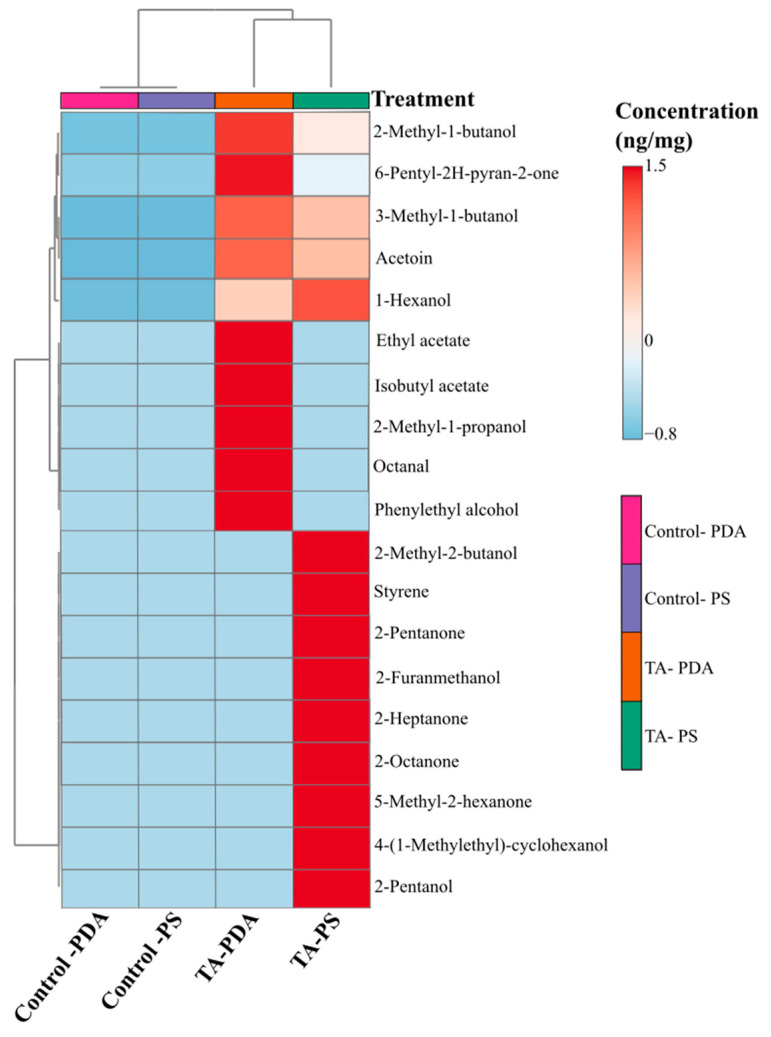
Heat map analysis with hierarchical cluster analysis (HCA) of the fungal volatile organic compounds (FVOCs) collected from *Trichoderma atroviride* (TA: isolate 1416) and a control (fungus-free) treatment (n = 10). Fungal volatiles were collected for 24 h from treatments grown on two different types of media: (1) potato–dextrose agar (PDA), and (2) mountain pine beetle diet media (PS: PDA with phloem and sapwood powder). The colour gradient represents the relative abundance of each chemical identified from high (darkest red) to low (darkest blue). The Euclidean distance measure and Ward clustering method were used in the analysis.

We quantified 30 compounds detected from the headspace volatile collection of *T. atroviride* grown on two different media types: (1) PDA, and (2) MPB diet media containing PDA mixed with phloem and sapwood powder (PS). From the 30 chemicals quantified, 19 were found to be previously identified FVOCs released by *T. atroviride* and other fungal species: 10 identified from the PDA media treatment and 14 from the MPB diet media treatment (Figure 3).

The *T. atroviride* profile revealed some similarities with the FVOCs identified from the headspace volatile collection of MPB symbiotic fungi *O. montium, G. clavigera*, and *L. longiclavatum* grown on lodgepole pine phloem by Zaman et al. (2023) [6]. *Trichoderma atroviride* released relatively high concentrations of four volatiles also identified for the symbiont species: 2-methyl-1-butanol, 2-methyl-2-butanol, 3-methyl-1-butanol, and acetoin.

We observed differences in the concentration and composition of volatiles collected from *T. atroviride* grown on both media types: 2-methyl-1-butanol, 6-pentyl-2H-2-one, 3-methyl-1-butanol, acetoin, and 1-hexanol were the only compounds identified from *T. atroviride* grown on both PDA and MPB diet media. The five most abundant volatiles identified from *T. atroviride* grown on PDA media were 3-methyl-1-butanol, 2-methyl-1-propanol, 2-methyl-1-butanol, 6-pentyl-2H-pyran-2-one, phenylethyl alcohol. Other than 2-methyl-1-butanol and 3-methyl-1-butanol, the five most abundant FVOCs released by *T. atroviride* grown on MPB diet media differed and included: 2-pentanone, 2-pentanol, and 2-heptanone. Generally, from the FVOCs identified from both media treatments, we observed higher mean volatile concentrations from *T. atroviride* grown on the PDA media (Table 3)

### 3.2. Mountain Pine Beetle Are Strongly Repelled by the FVOCs of T. atroviride

**Figure 4:**
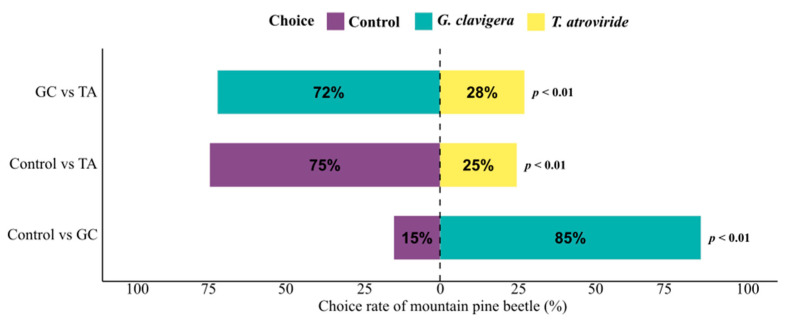
Choice rate of mountain pine beetles in two-choice olfactometer bioassay testing their attraction to fungal volatiles. The beetle preference for volatiles from one species of ophiostomatoid fungi, *Grosmannia clavigera* (GC), and one species of saprophytic fungi, *Trichoderma atroviride* (TA), as well as a fungus-free control treatment, was tested in unique treatment combinations.

In two-choice olfactometer assays, we were able to identify significant beetle preferences (*p* < 0.01) in each unique treatment combination tested (Table 4). The results of our olfactometer runs testing beetle preference for *G. clavigera* over the fungus-free control were consistent with previous findings by Zaman et al. (2023) [6]. The earlier study showed that MPBs strongly preferred FVOCs released by *G. clavigera* (80% beetle choice) over the control treatment. When testing *T. atroviride* against the fungus-free control, we found that 75% of beetles chose the control over the fungal treatment (*p* = 0.002) (Figure 4). Similarly, 72% of beetles chose *G. clavigera* over *T. atroviride* in olfactometer runs combining the two fungal treatments (*p* = 0.004).

### 3.3. Fungal Volatiles Did Not Strongly Influence MPB Feeding Behaviour

**Figure 5:**
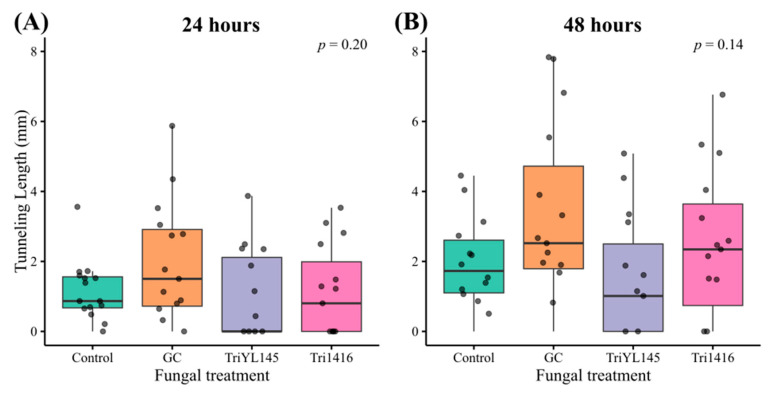
Boxplots comparing total mountain pine beetle tunnelling lengths (mm) in media exposed to different fungal volatile treatments after 24 (**A**) and 48 h (**B**). Fungal treatments included: (1) *Grosmannia clavigera* (GC), (2) *Trichoderma atroviride* isolate YL145 (TriYL145), (3) *T. atroviride* isolate 1416 (Tri1416), and (4) a fungus-free control. Note: Data from beetles that did not tunnel were included in this analysis. Boxes represent the interquartile range (IQR) of the data and horizontal lines inside the boxes show the median values. Whiskers extend to values 1.5 times from the IQR and points represent individual data points. Kruskal–Wallis rank-based tests were used to test for significance between treatments at 24 and 48 h.

**Figure 6:**
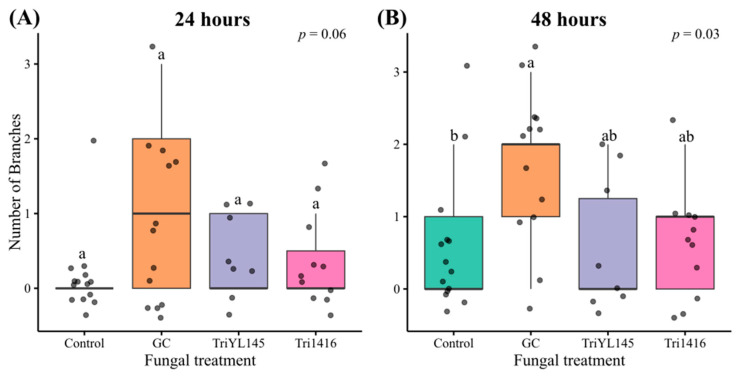
Boxplots comparing the number of branches coming off the longest tunnelling segment created by mountain pine beetles feeding in diet media while exposed to different fungal volatile treatments after 24 (**A**) and 48 h (**B**). Fungal treatments included (1) *Grosmannia clavigera* (GC), (2) *Trichoderma atroviride* isolate YL145 (TriYL145), (3) *T. atroviride* isolate 1416 (Tri1416), and (4) a fungus-free control. Data from beetles that did not tunnel were excluded from this analysis. Boxes represent the interquartile range (IQR) of the data, and horizontal lines inside the boxes show the median values. Whiskers extend to values 1.5 times from the IQR, and points represent individual data points. Different letters denote statistically significant differences between treatments based on results from Kruskal–Wallis rank-based tests and Dunn’s post hoc tests with Bonferroni’s *p*-value adjustment to control for multiple comparisons.

In our feeding bioassay experiment, we observed a large variation in beetle tunnelling measures within each treatment. Beetles feeding in the presence of volatiles from *T. atroviride* isolate YL145 had the lowest median total tunnelling lengths at both 24 and 48 h (Figure 5). However, we found no evidence of statistically significant effects on beetle tunnelling length at 24 or 48 h for any of the treatments (*p* > 0.05, Table 5). The number of branches coming off the longest tunnelling segment was the only variable found to differ significantly between treatments (*p* = 0.03). Tunnels created by beetles feeding in bioassay chambers containing FVOCs from *G. clavigera* had higher median numbers of branches after 48 h compared to the control treatment (*p* = 0.043, Figure 6). The number of branches after 24 h was also highest for the *G. clavigera* treatment; however, these results were found to be marginally non-significant (*p* = 0.056) compared to other treatments. Pairwise comparisons between treatments other than *G. clavigera* and the control revealed no significant differences. Approximately 37% of beetles in the *Trichoderma* treatments did not tunnel, compared to 13% and 7% in the *G. clavigera* and control treatments, respectively. However, these differences between treatments were also found to be statistically insignificant (*p* = 0.067).

## 4. Discussion

### 4.1. Volatiles Identified from Trichoderma Atroviride

Our analyses of the FVOCs produced by *T. atroviride* revealed several compounds with the potential to repel MPBs and suppress the growth of its symbiotic fungi. These FVOCs likely served a dual role in relation to the MPB host colonization process: directly deterring attraction of MPB adults, as observed in olfactometer tests, and once inside the host, indirectly affecting MPB fitness by inhibiting the growth of MPB symbiotic fungi.

Consistent with earlier findings [6], we observed both overlap and divergence between the FVOC profiles of *T. atroviride* and MPB-associated symbiotic fungi such as *G. clavigera* (Table 6). Notably, *T. atroviride* emitted high concentrations of 2-methyl-1-butanol, 2-methyl-2-butanol, 3-methyl-1-butanol, and acetoin—all among the five most abundant volatiles released by MPB symbionts, except for isobutanol [6]. The attraction of MPB, its predators, and competitors to each of these FVOCs has already been tested in field experiments [7]. Overall, 2-methyl-1-butanol, 2-methyl-2-butanol were identified as key FVOCs, possibly synergizing MPB attraction to its aggregation pheromones. This overlap may indicate that MPBs use these compounds as general cues for fungal colonization, rather than specific signals for beneficial or harmful fungal associates. These volatiles may also contribute to MPB’s ability to differentiate among fungal species through synergistic interactions with other compounds.

We were able to identify several FVOCs from the *T. atroviride* profile, which have previously been found to elicit behavioural responses in several insect species, two of which significantly impacted MPB attraction in the field [17,18,19,25,26]. One compound of particular interest is phenylethyl alcohol, which was detected in the *T. atroviride* profile and is known to reduce MPB attraction to pheromones [25,26]. This compound is also present in *L. longiclavatum*, which has been shown to repel MPBs in behavioural assays [6], suggesting phenylethyl alcohol may contribute to repellency in both species.

Green leaf volatiles are considered a promising group of chemicals for the development of anti-aggregants for bark beetles, by disrupting the ability of beetles to differentiate between host and non-host species [18]. Two green leaf volatiles (GLVs)—1-hexanol and 2-pentanol—were found in the *T. atroviride* profile. 1-hexanol has previously been shown to reduce MPB attraction when used in combination with verbenone [17,18,19]. While the behavioural effects of 2-pentanol on MPBs are not yet known, it has been shown to elicit responses in cerambycid beetles in field tests [54,55], indicating its potential relevance in bark beetle systems.

As a known biocontrol genus, *Trichoderma* sp. are effective competitors against plant pathogenic and wood-decay fungi due to their ability to produce cell wall-degrading enzymes and antimicrobial volatiles [56,57,58]. In our study, the FVOC profile of *T. atroviride* included several known anti-fungal and mycotoxic compounds, including pentyl-2H-pyran-2-one, octanal, and phenylethyl alcohol [41,51,59,60,61,62,63,64]. These antimicrobial properties may contribute to reducing MPB fitness by inhibiting the development of their symbiotic fungi within host galleries, although further research is needed to fully understand these interactions.

Based on our findings and previous research, we recommend four compounds from the *T. atroviride* FVOC profile for field testing as potential MPB anti-attractants: 2-pentanone, 2-heptanone, 2-pentanol, and phenylethyl alcohol. These compounds were among the most abundant volatiles detected in *T. atroviride* grown on MPB diet media. Both 2-pentanone and 2-heptanone have shown repellent effects in other beetle species [50]. 2-Pentanol has also been shown to affect attraction in *Anoplophora glabripennis* [54,55]. Additionally, phenylethyl alcohol’s capacity to reduce MPB responses to pheromones [25,26] makes it a promising candidate, especially since its potential synergy with verbenone remains unexplored.

In conclusion, *T. atroviride* produces a diverse set of bioactive volatiles that may both disrupt MPB behaviour and suppress fungal symbionts. These findings support further field-based evaluations of selected FVOCs as part of an integrated pest management strategy targeting MPB infestations.

### 4.2. Effects of FVOCs on MPB Attraction

Two-choice olfactometer assays revealed strong repellency of *T. atroviride* FVOCs to MPBs, both when tested against a fungus-free control and against the symbiotic fungus *G. clavigera*. Mountain pine beetles showed a clear preference for *G. clavigera*, suggesting they can differentiate between volatiles emitted by mutualistic vs. antagonistic fungi. These findings support the hypothesis that saprophytic fungi, such as *T. atroviride,* release volatiles that repel MPBs and highlight the potential of such FVOCs as anti-attractants for field applications.

The mechanisms underlying the observed repellency are likely complex and not attributable to a single compound. While individual volatiles, such as phenylethyl alcohol, verbenone, and 1-hexanol identified from *T. atroviride,* are known to reduce MPB attraction to aggregation pheromones (Table 1) [17,19], our olfactometer assays tested complete fungal profiles rather than isolated chemicals, making it difficult to isolate the effects of specific compounds. The strong behavioural responses observed suggest that the overall chemical composition of *T. atroviride* volatiles—potentially including synergistic interactions—plays a critical role. These findings are consistent with prior studies demonstrating that bark beetle responses depend on both the blend and dosage of semiochemicals [65,66,67,68].

Indeed, dose-dependency has been documented in MPB responses to several compounds, including *trans*-verbenol, *exo*-brevicomin, and frontalin, where attractiveness often peaks at intermediate release rates and declines at higher doses [65,66,67,68]. In the case of *T. atroviride*, the concentrations of volatiles may play a critical role. Volatile production in fungi reflects metabolic activity and growth rate, with faster-growing fungi typically emitting higher quantities [69,70]. As *Trichoderma* species are generally more rapidly growing than MPB-associated fungi like *G. clavigera* (Table 2) [71], *T. atroviride* likely produces higher concentrations of volatiles, which may exceed attraction thresholds and trigger repellent behaviour in MPBs.

In conclusion, the observed repellency of *T. atroviride* FVOCs to MPBs is likely driven by a combination of volatile identity, concentration, and synergistic effects. While the full FVOC profile may be necessary to achieve repellency, the individual effects of compounds like phenylethyl alcohol and 1-hexanol warrant further investigation. These findings justify field testing of both individual and blended FVOCs as candidate anti-attractant compounds in MPB integrated pest management.

### 4.3. Effects of FVOCs on MPB Feeding

In our feeding bioassays, FVOCs released by both symbiotic and saprophytic fungal species did not have a statistically significant effect on MPB tunneling length. However, biologically meaningful trends were observed. Beetles exposed to *T. atroviride* (YL145) demonstrated the lowest median tunneling lengths after 24 and 48 h, whereas those exposed to *G. clavigera* exhibited the highest tunneling lengths. This suggests that MPB feeding activity may be enhanced by volatiles from their symbionts and reduced in the presence of volatiles from antagonistic fungi like *T. atroviride*.

These trends also imply that MPBs may respond differently to FVOCs based on the ecological role of the emitting fungus. Although statistical significance was not achieved, the consistent reduction in feeding under *T. atroviride* exposure suggests a potential inhibitory effect of these volatiles on MPB behaviour. To our knowledge, no previous studies have examined the specific impact of fungal volatiles alone on MPB feeding. However, several studies have demonstrated that the presence of fungi can influence bark beetle tunneling behaviour. For example, Kandasamy et al. (2023) [27] reported increased tunneling by *Ips typographus* in diet media amended with host tree monoterpenes and colonized by symbiotic fungi. They suggested this was due to the production of oxygenated monoterpenes by the fungi, which are known to influence beetle behaviour and potentially act as feeding stimulants.

In our study, fungi were grown on media lacking host-derived monoterpenes, which may have limited their ability to produce oxygenated volatiles and, in turn, affected MPB feeding responses. This methodological difference could explain the absence of significant effects in our assays. Nonetheless, the observed trends indicate that FVOCs from fungi, even without monoterpene precursors, may still influence MPB behaviour, and future studies should explore how host–fungus chemical interactions shape feeding.

Interestingly, beetles exposed to *G. clavigera* volatiles created significantly more branching segments after 48 h compared to those in the fungus-free control. However, no significant difference in branching was found between *G. clavigera* and *T. atroviride* treatments. Hofstetter et al. (2006) [29] observed that *D. frontalis* larvae created longer tunnels when avoiding antagonistic fungi, implying that shorter and more complex branching could indicate reduced stress or more exploratory feeding under favourable conditions. Nevertheless, the ecological significance of branching behaviour in adult MPBs remains unclear and has not been rigorously quantified in prior bioassays.

In summary, although no significant differences in MPB feeding were detected in response to fungal volatiles, our findings point to meaningful behavioural trends: increased tunneling and branching in response to symbiotic fungi and decreased feeding in response to saprophytic antagonists. These results highlight the importance of considering volatile identity, concentration, and potential synergistic effects in future research. Testing fungal volatiles derived from monoterpene-amended media may help further clarify how host–fungus interactions influence MPB feeding behaviour and could inform semiochemical-based management strategies.

### 4.4. Study Limitations

This study has some potential limitations. First, our chemical analyses were conducted using only a single isolate of *T. atroviride*. Relying on one isolate may limit the reproducibility of our findings and may not fully capture the natural variability in FVOC production among different *T. atroviride* isolates. Since volatile profiles may vary significantly between isolates, this could affect the generalizability of our results. That said, many of the FVOCs we identified have also been reported in previous studies involving multiple *Trichoderma* isolates (Table 6), suggesting some consistency across strains. Second, we did not evaluate whether the candidate FVOCs elicit physiological responses in MPBs or related subcortical insects. Specifically, we were unable to perform electroantennography (EAG) to determine if MPB antennae can detect these compounds. Future research should include EAG assays to confirm the beetles’ ability to perceive the identified volatiles before advancing to field trials.

## 5. Conclusions

MPBs interact with various microbes in the host tree, including beneficial symbiotic fungi and antagonistic saprophytic fungi like *T. atroviride*. While symbiotic fungi can attract MPBs through specific FVOCs such as 2-methyl-1-butanol and 2-methyl-2-butanol—which may enhance beetle attraction at low population densities [6,7]—saprophytic fungi like *Trichoderma* and *Aspergillus* have been shown to reduce MPB fitness by altering its habitat and outcompeting symbionts [1,4]. Despite growing knowledge of symbiont-related FVOCs, less is known about the volatiles produced by antagonistic fungi. This study addressed that gap by characterizing FVOCs from *T. atroviride* and testing their effects on MPB behaviour. The results showed strong MPB repellency and only mild effects on feeding. Four compounds—2-pentanone, 2-heptanone, 2-pentanol, and phenylethyl alcohol—were identified as promising anti-attractants for future field testing. These findings suggest that saprophytic fungal volatiles could enhance MPB management strategies, particularly in push–pull systems targeting low-density populations. Future work should explore their standalone and synergistic potential with known repellents like verbenone in field conditions.

## Figures and Tables

**Table 1:** Summary of anti-attractant compounds and blends found to reduce attraction of mountain pine beetles to their pheromones. Synthesized using Progar et al. (2013) [16].

	Anti-Attractant Compounds	Source of Compounds	Function	References
**Non-host volatiles**	Verbenone with a blend of non-host volatiles (1-Hexanol, E-2- & Z-3-hexen-1-ol and benzyl alcohol)	Non-host volatiles from aspen bark	Significantly reduced catches on baited trees and funnel traps. Did not affect clerid predators.	[17,18,19]
**Con-specific volatiles**	Verbenone	MPBs	Most effective known anti-attractant for MPBs and other bark beetle species. Mixed results in some applications.	[16,20,21,22,23]
	1-Octen-3-ol	Female MPBs	Decreased response to pheromones.	[24]
	Phenylethyl alcohol	Male MPBs	Decreased response to pheromones.	[25,26]
**Hetero-specific volatiles**	3-methyl-2-cyclohexen-1-one	Anti-aggregation pheromone of *D. pseudotsugae* and *D. rufipennis*	Decreased response to pheromones.	[24]

**Table 2:** Description of fungal isolates used in our experiments and their respective growing times. DNA sequencing from isolates was used to confirm identity of each species. Each isolate was grown on mountain pine beetle (MPB) diet media and potato–dextrose agar (PDA) media until mycelium occupied 70% of petri dish before being used in our experiments. *Trichoderma atroviride* isolate 1416 was provided by Northern Forestry Centre (NFC) collection (Edmonton, AL, Canada).

Isolate Source	Isolate Label	Species	Identity	Growth on PDA (Days)	Growth on Diet Media (Days)
NFC	*Trichoderma* 1416	*T. atroviride*	100%	3	4
Lodgepole pine phloem	*Trichoderma* YL145	*T. atroviride*	100%	3	4
Lab collection	*Grosmannia Clavigera* EL035	*G. clavigera*	100%	N/A	5

**Table 3:** Mean concentrations (ng/mg) for headspace volatiles collected from *Trichoderma atroviride* (isolate 1416) grown on two different media types: (1) potato dextrose agar (PDA, n = 10), and (2) mountain pine beetle diet media containing PDA mixed with phloem and sapwood powder (PS, n = 10). Volatiles were collected for 24 h. All listed chemicals are volatiles that have been previously identified from fungi.

Volatile Compounds	PDA	PS
	Mean (SE)	Mean (SE)
2-Methyl-1-butanol	375.06 (92.5)	21.71 (3.7)
3-Methyl-1-butanol	1126.21 (325.4)	343.25 (55.8)
Acetoin	38.92 (8.9)	10.95 (3.9)
1-Hexanol	2.12 (1.2)	3.37 (1.3)
6-Pentyl-2H-pyran-2-one	167.29 (35.6)	2.37 (1.2)
Ethyl acetate	33.59 (8.1)	0.00 (0.0)
2-Methyl-1-propanol	691.99 (122.7)	0.00 (0.0)
Isobutyl acetate	11.97 (2.9)	0.00 (0.0)
Octanal	0.81 (0.5)	0.00 (0.0)
Phenylethyl alcohol	83.58 (17.1)	0.00 (0.0)
2-Methyl-2-butanol	0.00 (0.0)	1.38 (0.7)
Styrene	0.00 (0.0)	0.61 (0.4)
2-Pentanone	0.00 (0.0)	46.21 (6.9)
2-Furanmethanol	0.00 (0.0)	4.19 (1.2)
2-Heptanone	0.00 (0.0)	16.11 (2.5)
2-Octanone	0.00 (0.0)	0.75 (0.2)
5-Methyl-2-hexanone	0.00 (0.0)	0.58 (0.2)
4-(1-Methylethyl)-cyclohexanol	0.00 (0.0)	5.41 (2.1)
2-Pentanol	0.00 (0.0)	17.74 (3.5)

**Table 4:** Results of chi-square goodness of fit tests for mountain pine beetle (MPB), with preference for fungal volatile treatments in two-choice olfactometer assays. Degrees of freedom (Df), chi-squared statistic ($X^{2}$) and *p*-values are shown. Treatments were fungal isolates grown on MPB diet media: (1) *Trichoderma atroviride* isolate YL145, (2) *T. atroviride* isolate 1416, (3) *Grosmannia clavigera* isolate EL035, and (4) a fungus-free control. Each unique combination of the four treatments was tested 20 times. A 6 mm plug of treatment was placed on either side of the olfactometer setup and beetle choice was recorded after 20 min. Results from the two *T. atroviride* isolates were combined for statistical analyses (n = 40).

Treatment Combination	Df	X 2	*p*-Value
Control vs. *G. clavigera*	1	9.8	0.002 *
Control vs. *T. atroviride*	1	10	0.002 *
*G. clavigera* vs. *T. atroviride*	1	8.1	0.004 *

* *p* < 0.01.

**Table 5:** Results from the Kruskal–Wallis rank-based test for significant differences (*p* < 0.05) between fungal volatile treatments for each indicator of mountain pine beetle feeding activity measured. The table includes degrees of freedom (Df), chi-squared statistic ($X^{2}$), and *p*-values from each test. Note: Data from beetles that did not tunnel were excluded from the total tunnelling length analysis. The number of branches refers to the number of branches from the longest continuous tunnelling segment.

Time	Measurement	Df	X 2	*p*-Value
**24 h**	Number of branches	3	7.55	0.06
	Longest tunnel segment (mm)	3	0.88	0.83
	Total tunneling length (mm)	3	4.63	0.20
**48 h**	Number of branches	3	9.09	0.03 *
	Longest tunnel segment (mm)	3	4.36	0.23
	Total tunneling length (mm)	3	5.43	0.14

* *p* < 0.05.

**Table 6:** Summary of fungal volatiles (FVOCs) identified from *Trichoderma atroviride* headspace volatiles and behavioural responses they are known to elicit in subcortical insects. Volatiles were collected from fungi grown on two different media types: (1) potato dextrose agar (PDA), and (2) mountain pine beetle (MPB) diet media containing PDA mixed with phloem and sapwood powder (PS). Note: All listed chemicals have been previously identified as FVOCs.

Identified FVOCs	Present in PDA	Present in PS	Previous Findings	References
2-Methyl-1-butanol	x	x	Detected from MPB symbiotic fungi. Synergized attraction of MPB to its aggregation pheromones. Detected from *Trichoderma* sp. Inhibited wood decay fungal growth at high concentrations.	[6,7,39]
6-Pentyl-2H-pyran-2-one	x	x	Detected from FVOC of *Trichoderma atroviride.* Inhibited growth of several phytopathogenetic fungi.	[40,41]
3-Methyl-1-butanol	x	x	Detected from *T. atroviride.* Detected from MPB symbiotic fungi.	[6,13]
Acetoin	x	x	Detected from *T. atroviride.* Detected from MPB symbiotic fungi.	[6,13]
1-Hexanol	x	x	Detected from of *Trichoderma* sp. Antennal response in MPBs. When included in a blend of GLVs, it acted as an anti-attractant for MPBs and *Ips typographus.*	[17,18,33,42,43]
Phenylethyl alcohol	x		Decreased MPB response to pheromones. A pheromone component of *I. typographus.* Acetate ester 2-phenylethyl acetate produced by several bark beetle symbionts and found to attract *Dendroctonus frontalis.* Decreased *D. frontalis* attraction to pheromones. Highest concentration in the FVOC profile of *Leptographium longicalvatum* compared to other MPB symbiotic fungi.	[6,25,26,28,44,45,46,47]
2-Methyl-2-butanol		x	Detected from MPB symbiotic fungi.	[6]
Styrene		x	Strong antennal response in *I. typographus.* Induced defense chemical of *Picea abies.* Extracted from *Penicillium expansum* found in *Hylobius abietis* frass and reduced *H. abietis* attraction to host tissues.	[47,48]
2-Pentanone		x	Component of defensive secretions from *Uloma tenebrionoides.* Reduced *Sitophilus granarius* orientation towards food source (wheat grains).	[49,50]
2-Heptanone		x	Produced in trace amounts by male *Anaglyptus mysticus* (Cerambycidae) beetles. Reduced *S. granarius* orientation towards food source (wheat grains). Alarm pheromone in various insects, including ants.	[50,51,52]
2-Octanone		x	Volatile from *Rahnella aquatilis* bacteria isolated from *H. abietis* guts with antifeedant properties for the beetle.	[53]
2-Pentanol		x	Green leaf volatile and increased *Anoplophora glabripennis* attraction to pheromones in field experiments.	[54,55]

## Data Availability

The authors will make the raw data supporting this article’s conclusions available upon request.
